# Level of HIV serodiscordance and associated factors among heterosexual couples in Ethiopia: A systematic review and meta-analysis

**DOI:** 10.1371/journal.pgph.0003090

**Published:** 2024-06-20

**Authors:** Dagne Deresa Dinagde, Hana Tadesse Afework, Habtamu Wana Wada, Meserat Workiye Negash

**Affiliations:** 1 Departments of Midwifery, College of Health Sciences, Mattu University, Mettu, Ethiopia; 2 Departments of Midwifery, College of Health Sciences, Mizan Teppi University, Mizan Teppi, Ethiopia; 3 Departments of Midwifery, College of Medicine and Health Sciences, Arba Minch University, Arba Minch, Ethiopia; 4 Departments of Public Health, College of Medicine and Health Sciences, University of Gondar, Gondar, Ethiopia; University of Embu, KENYA

## Abstract

HIV-related causes accounted for approximately 770,000 deaths globally in 2018. Globally, there were 1.7 million new infections, and approximately 37.9 million people were living with HIV by the end of 2018. According to the WHO 2018 study, the African Region was the most affected, with 25.7 million people living with HIV in 2018. In Africa, married and cohabiting couples have a high prevalence of HIV discordance, ranging from 3% to 20% in the general population. Therefore, it is crucial to understand the level of HIV serodiscordance among married couples in Ethiopia and the contributing factors. Studies were systematically searched, utilizing international databases such as PubMed, Google Scholar, Cochrane Library, and Embase. The level of quality of the included articles, which employed cross-sectional and cohort study designs, was evaluated using the New Castle Ottawa scale. The systematic review employed a random-effects approach, and statistical analysis was conducted using STATA version 17 software. The presence of statistical heterogeneity within the included studies was assessed using the I-squared statistic. The random-effects meta-analysis model was used to estimate the pooled level of HIV serodiscordance. The results were reported following the Preferred Reporting Item for Systematic Review and Meta-Analyses (PRISMA) guideline. A total of ten (10) observational studies were included in this review. The pooled level of HIV serodiscordance among married heterosexual couples in Ethiopia was found to be 11.4% (95% CI = 7% -15.7%). The results from the meta-analysis indicated a significant positive association between HIV serodiscordance and the variables studied. Specifically, consistently using condoms (OR = 1.82; 95% CI: 1.08–2.56), having a CD4 count of >200 cells/mm3 (OR = 1.45; 95% CI: 1.12–1.77), and having a premarital sexual relationship (OR = 1.93; 95% CI: 1.28–2.57) were strongly linked to couples’ serodiscordance. To protect a seronegative partner in a serodiscordant relationship from acquiring HIV infection, it is crucial to implement preventive measures. These measures include providing comprehensive health education on the correct and consistent use of condoms, ensuring regular monitoring and care at an antiretroviral therapy (ART) clinic, and offering voluntary counseling and testing (VCT) services to both sexual partners.

## Background

HIV attacks immune system cells, which are the body’s first line of defense against diseases. Consequently, the body becomes less capable of combating infections and certain types of cancer. The virus targets and destroys CD4 cells, a type of white blood cell in the immune system, while replicating itself within these cells. HIV has claimed the lives of over 35 million individuals globally, making it a significant public health concern [1]. Approximately 770,000 deaths worldwide were caused by HIV in 2018. By the end of that year, there were approximately 37.9 million people living with HIV globally, and 1.7 million new infections occurred. According to the WHO 2018 report, the African Region was the most heavily affected, with 25.7 million people living with HIV in 2018. Nearly two-thirds of all new HIV infections worldwide occur in this region. In 2018, around 1.1 million people in the African Region were newly infected with HIV [2].

HIV-discordant couples are characterized by one partner being HIV infected while the other partner is HIV-negative [3]. In such relationships, the risk of HIV transmission is higher within the committed partnership than from outside sexual partners. It is important to convey to couples that being in a committed and monogamous relationship does not eliminate the possibility of HIV transmission between partners [4]. Within the African population, there is a significant occurrence of HIV discordance among married and cohabiting couples, with prevalence rates ranging from 3% to 20% [5]. For instance, HIV serodiscordance in Uganda was 49.2% [6], 16% in Tanzania [7] and 1.8%–23.6% in different parts of Ethiopia [8–10]. In sub-Saharan Africa, around half of the individuals living as a couple with HIV-1 infection have a partner who is seroconcordance. Recent data indicates that a significant portion of new HIV-1 infections in well-established epidemics occur within such discordant couples, highlighting the role of discordance in the spread of HIV/AIDS in Africa. Less than 10% of HIV-positive individuals are aware of their partners’ status, and only approximately 20% of HIV discordant couples in East Africa are aware of being in a serodiscordant relationship [11–13].

Sero-conversion rates are lower in discordant couples who have undergone VCT and other therapies; however, the incidence in these couples is still significant, ranging from 3 to 8% each year [6,14]. Efforts to increase the effectiveness of couples VCT and other interventions for HIV-discordant couples in Africa may benefit from a thorough understanding of their experiences. Compared to non-regular discordant couples, transmission is higher in regular, established discordant partnerships [15]. Most discordant couples in Ethiopia live in urban areas. Of the 2,674 cohabiting couples in Ethiopia who had HIV tests performed in the DHS in 2005, 1.8% were discordant. 1% of married men without HIV live with their infected wives, and 0.8% of married women without HIV live with their infected husbands [16].

The WHO’s endorsement of U = U in 2018 marked a significant milestone, affirming that individuals with HIV on effective treatment can have sex without transmitting the virus. Regular viral load monitoring, adherence to ART, and maintaining an undetectable viral load are essential for U = U to apply. This concept is supported by strong scientific evidence, showing that those with HIV who achieve and sustain an undetectable viral load cannot sexually transmit the virus [17,18].

For clinical studies assessing the efficacy of vaccinations, microbicides, and other preventive strategies against HIV, HIV-discordant couples serve as a valuable cohort. Hence, it is crucial to comprehend the HIV serodiscordance status among married heterosexual couples or cohabiting individuals in order to mitigate the spread of HIV/AIDS in Ethiopia. Therefore, the objective of this systematic review and meta-analysis was to estimate the overall prevalence of HIV serodiscordance and identify factors associated with serodiscordance among couples in Ethiopia.

## Methods

### Study design and setting

To determine the pooled prevalence of HIV serodiscordance among married (cohabited) couples and related factors in Ethiopia, a systematic review and meta-analysis were undertaken. With an estimated 121 million people, Ethiopia is a landlocked nation on the Horn of Africa. Its neighbors include Sudan to the northwest, Eritrea to the northwest, Djibouti to the northeast, Somalia to the east, Kenya to the south, and Eritrea to the north [19].

### Reporting

The Preferred Reporting Items for Systematic Review and Meta-Analysis statement (PRISMA) guideline was used to report the review’s findings [20]. However, the study was not registered on PROSPERO, a prospective registry for systematic reviews and meta-analyses. This limitation was addressed in the section titled “Limitations of the Study.”

### Search strategies

Various databases were comprehensively searched, including PubMed, Embase, Science Direct, Cochrane Library, African Journals Online, Google Scholar, and Web of Science, to conduct an extensive literature search. The search was initiated using the full title (“HIV serodiscordance and associated factors among married couples in Ethiopia:”) and relevant keywords, such as “HIV,” “serodiscordance,” “predictors of HIV serodiscordance,” “associated factors of HIV discordance,” and “in Ethiopia.” These keywords were combined using Boolean operators “OR” or “AND,” and search terms were also created using Medical Subject Heading (MeSH) phrases, either individually or in combination. Or using full searching query for PubMed “("HIV serodiscordance"[All Fields] OR "HIV serodiscordant"[All Fields] AND ("heterosexual couples"[All Fields] AND ("associated factors"[All Fields] OR "risk factors"[All Fields] OR "determinants"[All Fields])”. To identify any additional relevant studies that may have been overlooked, the reference lists of all included studies were also examined. The literature search was conducted between November 10 and December 14, 2023.

### PECO

The Population, Exposure, Comparison, and Outcomes statement also used in this review; Population: (all married (cohabited) couples in Ethiopia; Exposure: determinants of HIV serodiscordance; Comparison: stated control group in each individual research; and Outcome: HIV serodiscordance (Yes/No).

### Eligibility criteria

#### Inclusion criteria

The authors utilized the PICO technique to establish the eligibility criteria for this systematic review and meta-analysis. The criteria mainly focused on Condition, Context, and Population (CoCoPop) questions for prevalence studies.

- *Study area*: study done in Ethiopia.
- *Study design*: Any observational paper or study that presents information on the frequency measures, such as incidence, prevalence, or size, of HIV serodiscordance, as well as papers that include both frequency measures and associated factors, will be included in the analysis.
- Studies with pertinent results that published in English were included for the purpose of simplicity and interpretive clarity.
  *Publication condition*: Both published and unpublished studies.
- *Time*: All studies conducted in Ethiopia were included, and there was no restriction placed on the date of publication.

#### Exclusion criteria

Studies that yielded a different outcome of interest were excluded from the analysis, as well as qualitative studies that did not provide quantitative data to support the combined estimate. Additionally, after making contact with the principal investigator through at least two emails, studies that used different operational definitions and assessments of the level of HIV serodiscordance were also excluded from this systematic review and meta-analysis.

### Data extraction

Using a piloted Microsoft Excel spreadsheet for data extraction, four reviewers (DD, HT, MW, and HT) independently assessed the relevance of each paper (initial screening), evaluated the full-text articles for eligibility (full-text screening), considered the study’s inclusion criteria and objectives (eligibility assessment), and then performed data extraction. The initial author’s name, publication year, study year, study area, sample size, and the proportion or level of HIV seroconcordance are all listed in the spreadsheet. Wherever there was dispute among the reviewers, it resolved through discussion and a new assessment of each study.

### Data quality assessment

The Newcastle-Ottawa Scale (NOS) quality assessment tool was used to assess the quality of included studies based on the three components [21]. The main section of the instrument assessed the methodological rigor of each primary study using a scale ranging from 1 to 5. The last component of the tool was scored using a three-star system to evaluate the results and statistical analysis of each original study. Another component of the tool was graded using a two-star system to express concerns regarding the comparability of each study. The Newcastle-Ottawa Scale (NOS) consists of three categorical factors, with a maximum score of 10. The quality of each study was assessed using the following scoring methods: a study with seven or more points was classified as "good" quality, two to six points as "fair" quality, and one point as "poor" quality [22] (Table 1).

**Table 1:** Summary of characteristics of included studies.

Authors & year of publication	Region	Study setting	Study design	Sample size	Magnitude	Quality assessment (score)
Tadesse M., 2014 [23]	SNNPR	IB	Cross-sectional	152	5.9%	6
Habte E. et al, 2015 [24]	Oromia	IB	Case-control	2370	8.4%	9
Asefa W. et al, 2006 [25]	Amhara	IB	Cross-sectional	601	9.8%	8
Elias G. et al, 2014 [9]	Oromia	IB	Cohort study	117	1.7%	4
Bantigen K. et al, 2019 [26]	Addis Ababa	IB	Cohort study	227	16.7%	6
Temam G.et al, 2012 [27]	Addis Ababa	IB	Cross-sectional	392	6.5%	7
Gedfew M. et al, 2018 [8]	Amhara	IB	Cohort study	212	23.6%	6
Barnabas G. et al, 2014 [28]	Tigray	IB	Sub-national level	61,000	16.4%	8
Cherinet Y. et al, 2013 [10]	Oromia	IB	Cross-sectional	1247	4.9%	9
Teshome GS. Et al,2021 [29]	Addis Ababa	IB	Cross-sectional	216	23.1%	5

IB = Institutional based, SNNPR South Nation Nationality of people Region.

### Measurement of the outcome of interest

The main finding of this systematic review and meta-analysis was the level of HIV serodiscordance among married or cohabiting couples in Ethiopia. The secondary outcome variable was the associated factors of HIV serodiscordance, which were estimated using a pooled adjusted odds ratio (AOR) with 95% confidence intervals (CIs). HIV serodiscordance was defined as a situation where one partner was living with HIV while the other partner was HIV-negative. The couples included in the study were either married individuals or individuals cohabiting without formal marriage, as reported by one of the couples and recorded in the charts [23,24].

### Statistical methods and analysis

The data imported from the Microsoft Excel spreadsheet was analyzed using the STATA 17 statistical software. For the current meta-analysis, the MetaAnalyst beta 3.3 software version was used to calculate the pooled prevalence of the overall level of HIV discordance in Ethiopia. The degree of study heterogeneity was assessed by calculating the I^2^ statistic, which represents the percentage of total variation among studies attributable to heterogeneity rather than chance. A score of 30% to 60% on the I2 statistic may indicate moderate heterogeneity, a value of 50% to 75% may indicate substantial heterogeneity, and a value of 75% to 100% may indicate significant heterogeneity [30]. Therefore, the pooled level of HIV serodiscordance was estimated using a random effects model utilizing the DerSimonian-Laird technique. The OR and 95% CI were constructed from the included studies.

#### Subgroup analyses and heterogeneity

To identify possible causes of heterogeneity amongst the included studies, subgroup analyses based on whether the participants were on antiretroviral therapy (ART) or not, depend on year of publication and quality of the study were conducted.

### Publication bias and heterogeneity

The funnel plot shows symmetrically distribution among individual studies indicating that there is no publication bias among the included studies. The overall heterogeneity of HIV serodiscordance was Ι^2^ = 98.76%, with P< 0.001 by use of the random effect model to adjust observed variability.

### Ethical statement

Not applicable; since we did not use primary data that needs ethical approval and consent to participate.

## Results

### Selection and identification of studies

**Fig 1:**
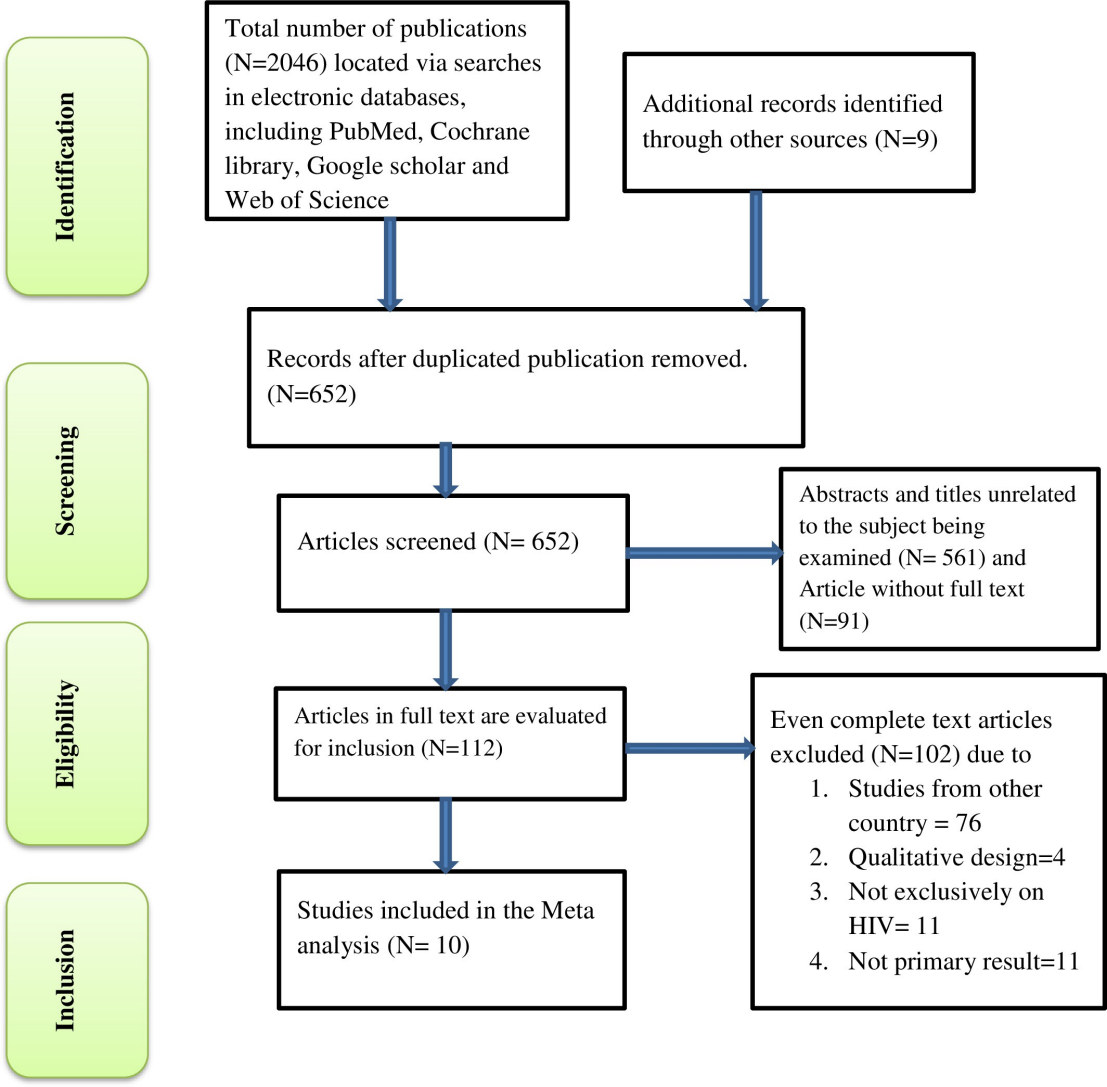
Flow chart diagram describing selection of studies included in the systematic review and meta-analysis using PRISMA checklist, 2023.

A total of 2046 published and unpublished studies were identified through a search of various worldwide databases and the institutional repositories. The studies were then screened using the EndNote reference manager, and duplicate studies were removed. As a result, 652 articles were subjected to abstract and title screening. From this screening process, 1394 articles were eliminated due to duplications and irrelevancies, leaving 112 complete papers for further evaluation. Subsequently, 102 studies were excluded based on various criteria, such as being conducted in a different country (76), having a qualitative design (4), not exclusively focusing on patients living with HIV (11), or not providing primary results on the desired outcome (11). Ultimately, a total of ten studies that met the inclusion criteria were included in the analysis for this study (Fig 1).

### Characteristics of the included studies

A total of 66,534 couples were included in this meta-analysis, with a 100% response rate. All the included studies were published in international journals between 2006 and 2023. From a total, ten (10) included studies; five were cross-sectional studies, three cohort studies and one case control study. The included studies had samples sizes ranging from 117 [9] to 61,000 [28]. All studies were conducted only in three regions and one administrative city of Ethiopia (Addis Ababa). The lowest prevalence was observed from study conducted at Jimma specialized hospital, Oromia regional state (1.7%) and the highest prevalence was noted from study conducted in Amhara region, 23.6% [8]. There were five articles of good quality according to the Newcastle Ottawa scale of quality assessment, with a score of seven or higher. Additionally, there were five articles of fair (moderate) quality, with scores ranging from two to five. As a result, half of the articles (5) had good quality assessment scoring ≥ 7. This critical evaluation was carried out to evaluate the studies’ internal validity (systematic error) and external validity (generalizability) and to lower the risk of bias (Table 1).

### The level of HIV serodiscordance among (cohabiting or married couples)

**Fig 2:**
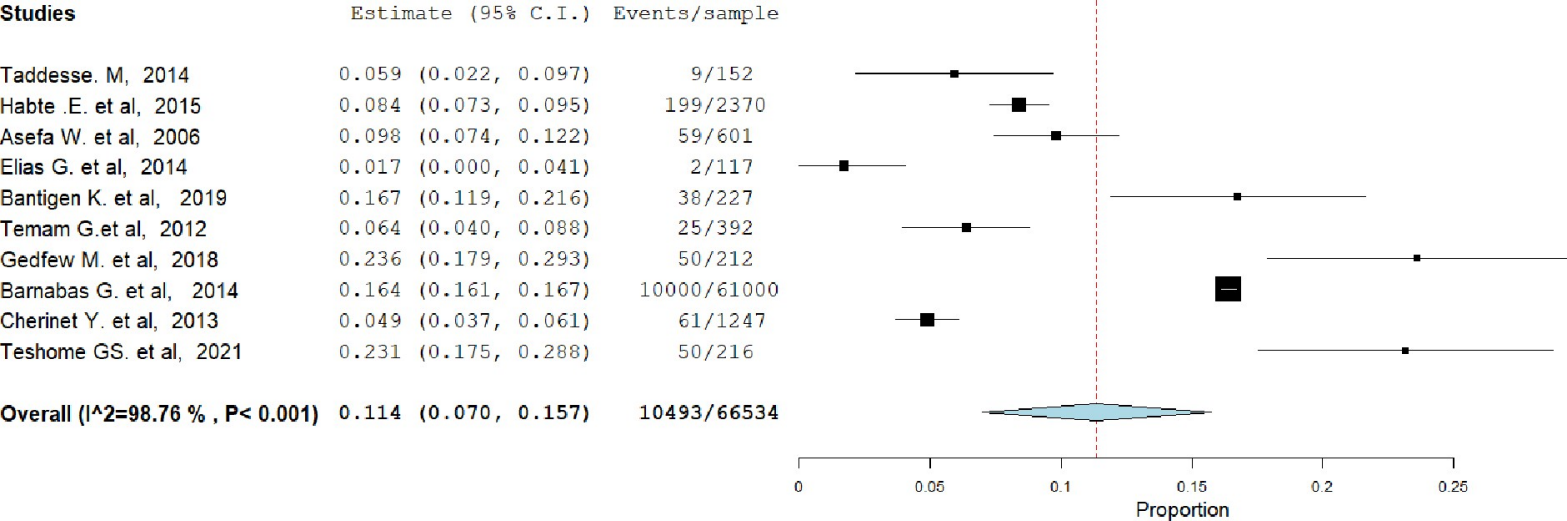
Proportion of sero-discordant couples in Ethiopia, 2023.

The overall pooled magnitude of HIV serodiscordance in Ethiopia was found to be 11.4% (95% CI = 7–15.7). Study heterogeneity was examined using the I2 test, and the results revealed that there was a significant degree of variation between the studies (I^2^ = 98.76%, P value < 0.001) (Fig 2).

#### Subgroup analysis

**Fig 3:**
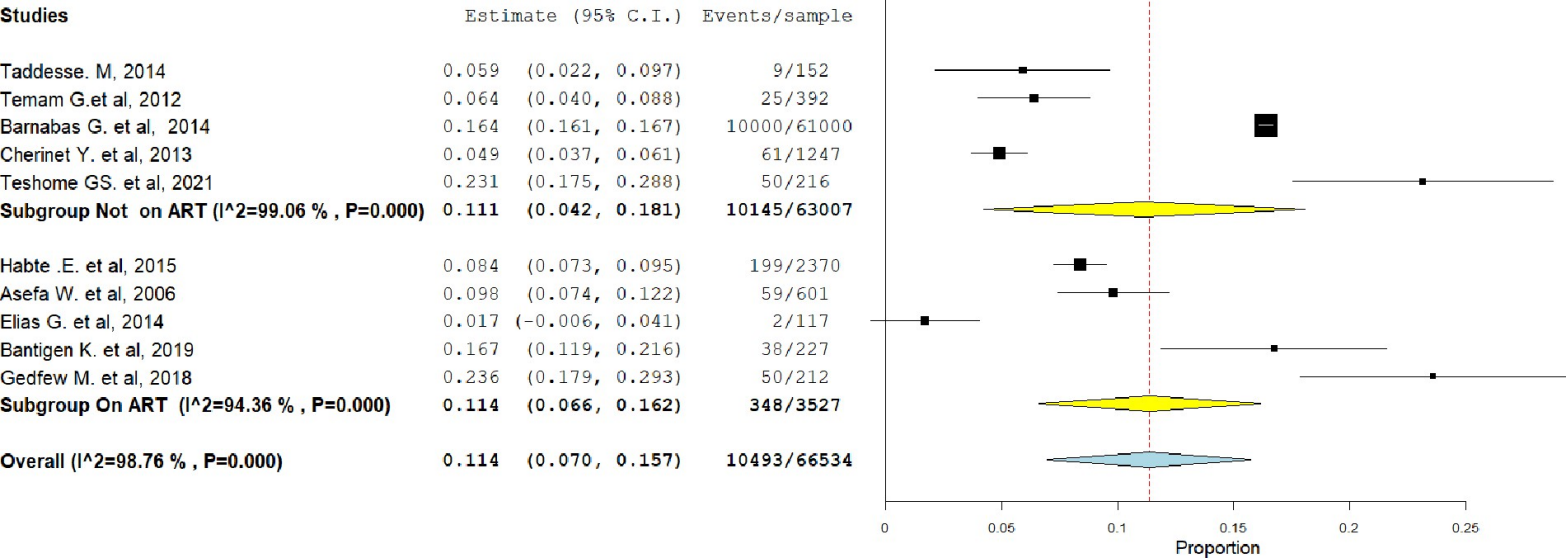
Sub-group analysis of studies included in meta-analysis depend on whether the individuals had been on ART or not in Ethiopia, 2023.

The pooled level of HIV serodiscordance was subjected to subgroup analysis depending on whether the individuals (couples) have been on antiretroviral drugs (ART) or not in order to determine the source of variability. Thus, Studies conducted on those who had been receiving antiretroviral drugs (ART) found a considerably higher serodiscordance among the couples (12.2%, 95% CI = 8.1, 16.2) (Fig 3). The level of heterogeneity appears to be greater among studies conducted on individuals who have not received antiretroviral therapy (ART) compared to studies conducted on individuals receiving ART.

**Fig 4:**
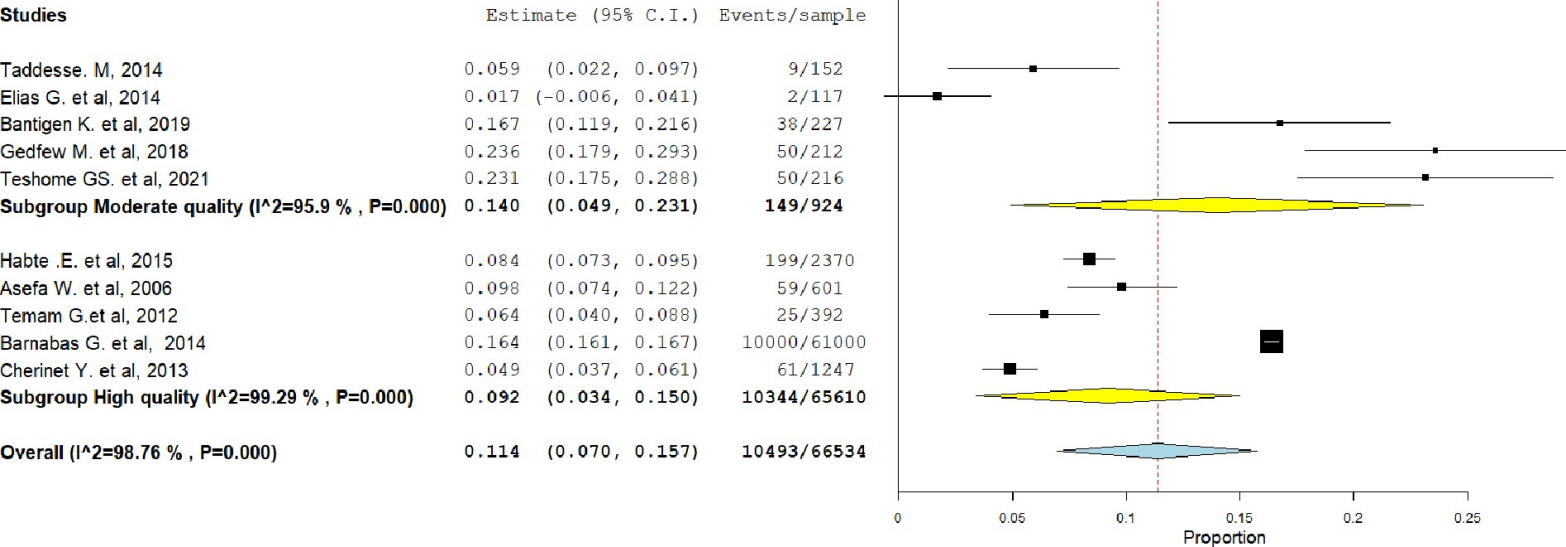
Sub-group analysis of studies included in meta-analysis depend on quality of studies in Ethiopia, 2023.

A subgroup analysis was conducted on the serodiscordance of HIV based on the quality of the included studies. The findings revealed that the overall prevalence of HIV seroconcordance was relatively higher among studies of moderate quality compared to those of good quality, 14%, (95% CI = 5%–23%) (Fig 4). The level of heterogeneity is greater among high-quality studies compared to moderate-quality studies.

**Fig 5:**
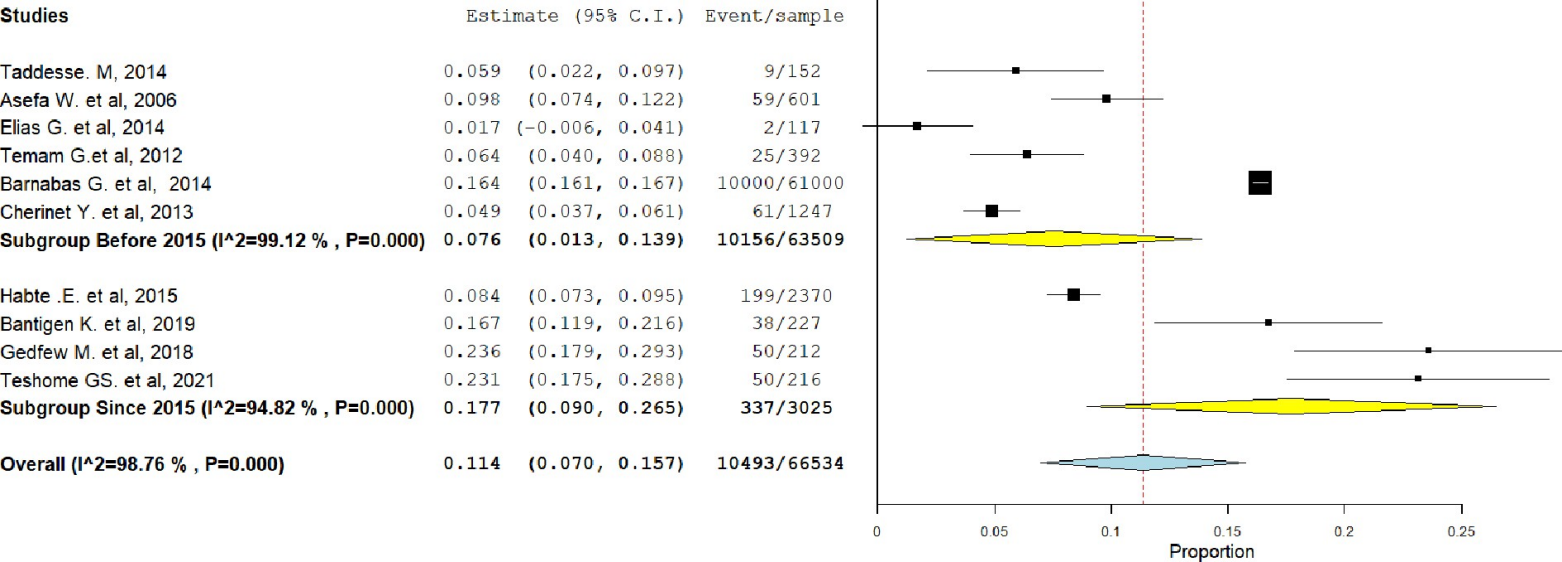
Sub-group analysis of studies included in meta-analysis depend year of publication, 2023.

In the subgroup analysis, the year of study conducted was utilized, as illustrated in (Fig 5). Among the included studies, the highest pooled prevalence of serodiscordance was observed in studies conducted from 2015 onwards, 17.7% (95% CI: 7%–15.7%). Greater heterogeneity was observed among studies conducted prior to 2015.

#### Sensitivity analysis

**Fig 6:**
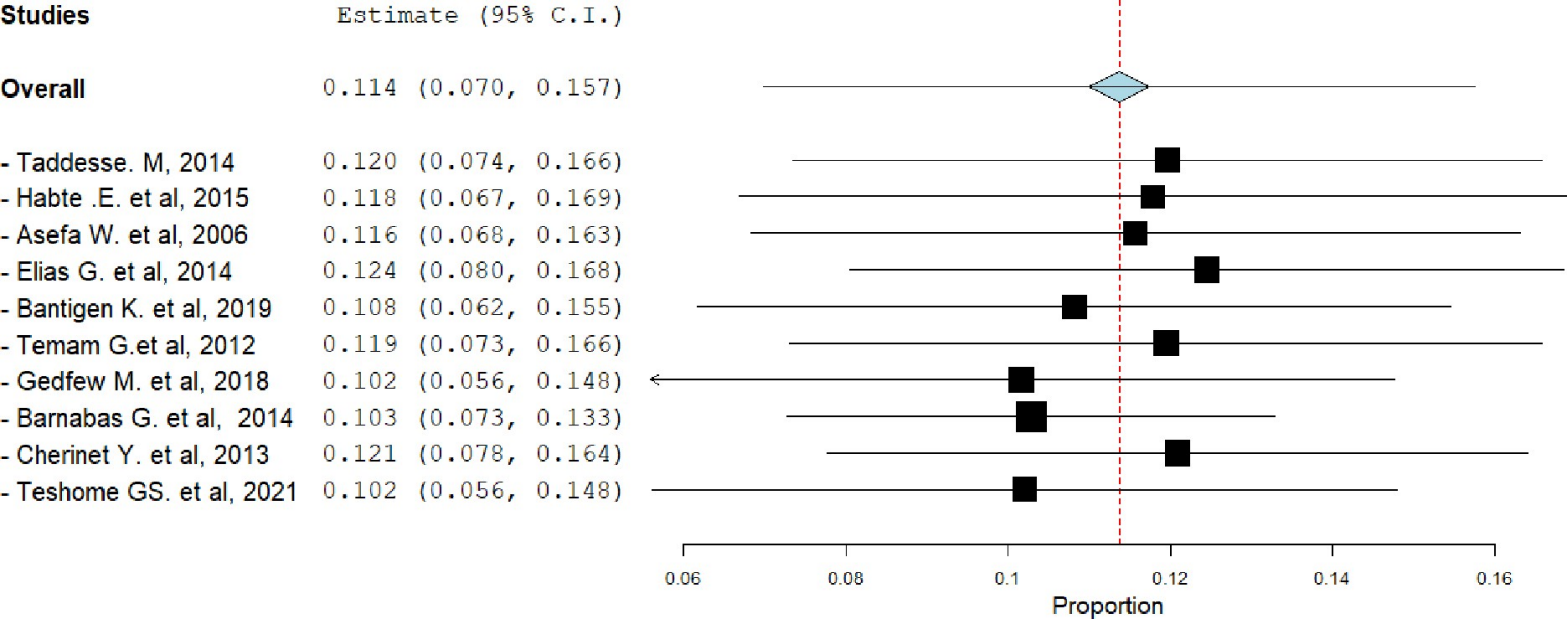
Proportion of HIV serodiscordance among heterosexual couples when one study omitted from the analysis a step at a time, 2023.

The researchers conducted a sensitivity analysis called leave-one-out, where they systematically excluded each study one at a time to see if it had a significant effect on the overall prevalence of HIV serodiscordance. The results of the analysis, using a random effect model, indicated that none of the individual studies had a significant impact on the prevalence of HIV serodiscordance infection among heterosexual couples (Fig 6).

#### Publication bias

**Fig 7:**
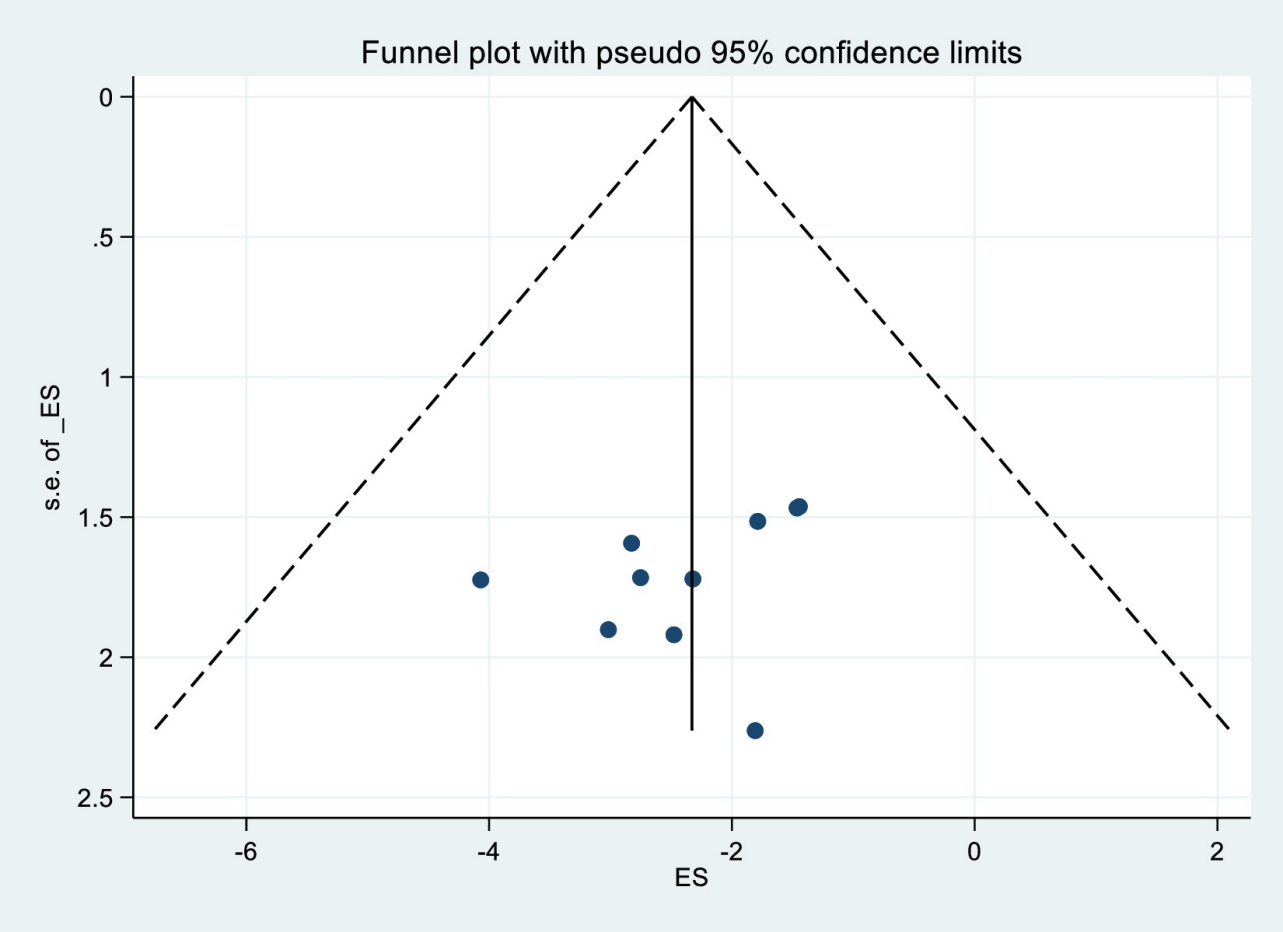
Funnel plot showing the study’s publication bias, 2023.

The analysis conducted on the HIV serodiscordance did not show any statistical evidence of publication bias. This was determined through the examination of a funnel plot and the use of the Egger regression test, with a significance level set at P > 0.05. The results of the Egger tests were not statistically significant, with P-values of 0. 0.084, indicating no significant bias. Additionally, the funnel plot displayed a symmetrical distribution, suggesting the absence of small-study effects or publication bias (Fig 7).

### Factors associated with HIV discordance

Due to inconsistent categorization or grouping of the exposures in relation to the outcome, some of the components related to HIV serodiscordance in this review were quantitatively pooled, while others were not. From the included studies, eight variables were considered to determine the major determinants of serodiscordance. Eventually, three variables (condom use, CD4 count, and premarital sexual practice) were identified as significant determinants of HIV serodiscordance.

**Fig 8:**
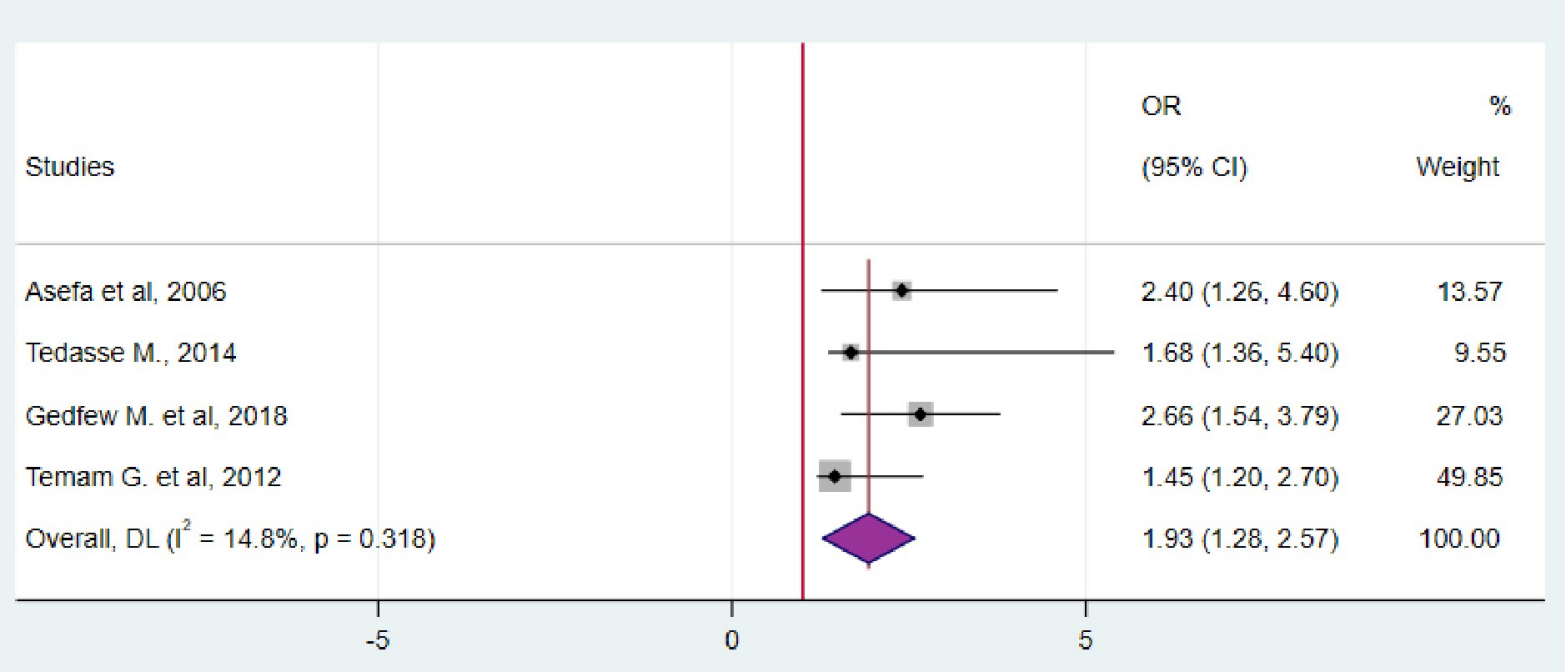
Forest plot for the association between premarital sexual relationship and HIV serodiscordance in Ethiopia, 2023.

Four studies indicated that couples who engaged in sexual intercourse before marriage were more likely to be serodiscordant compared to those who were cohabiting. The remaining studies included in the analysis did not show any association between the variables and the outcome. Consequently, our combined results demonstrated that couples who engaged in premarital sexual relationships were 93% more likely to be serodiscordant than their counterparts (OR = 1.93; 95%CI: 1.28–2.57) (Fig 8).

**Fig 9:**
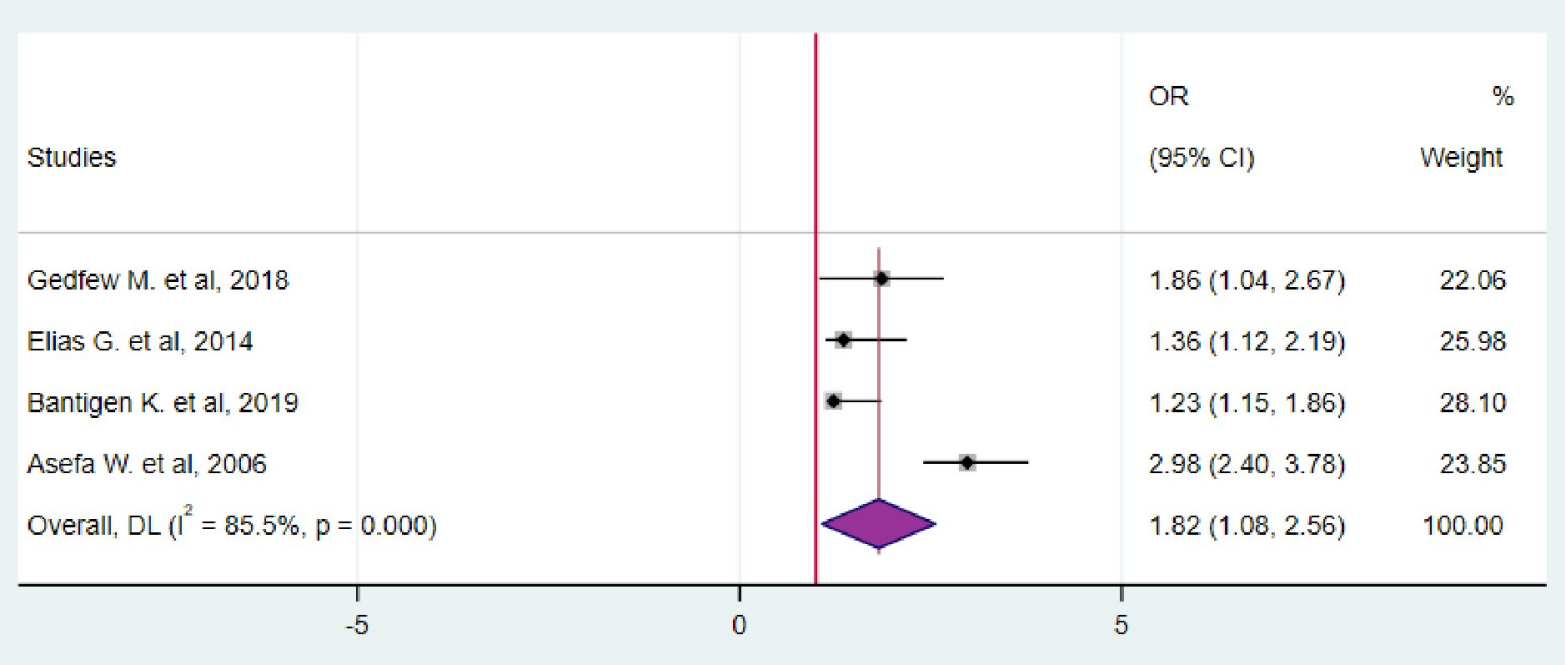
Forest plot for the association between regular condom use and HIV serodiscordance in Ethiopia, 2023.

Four studies evaluated the relationship between HIV serodiscordance and condom use. These studies showed that couples (or individuals) who regularly used condoms had an 88% higher chance of being HIV serodiscordance than those who did not (OR = 1.82; 95%CI: 1.08–2.56) (Fig 9).

**Fig 10:**
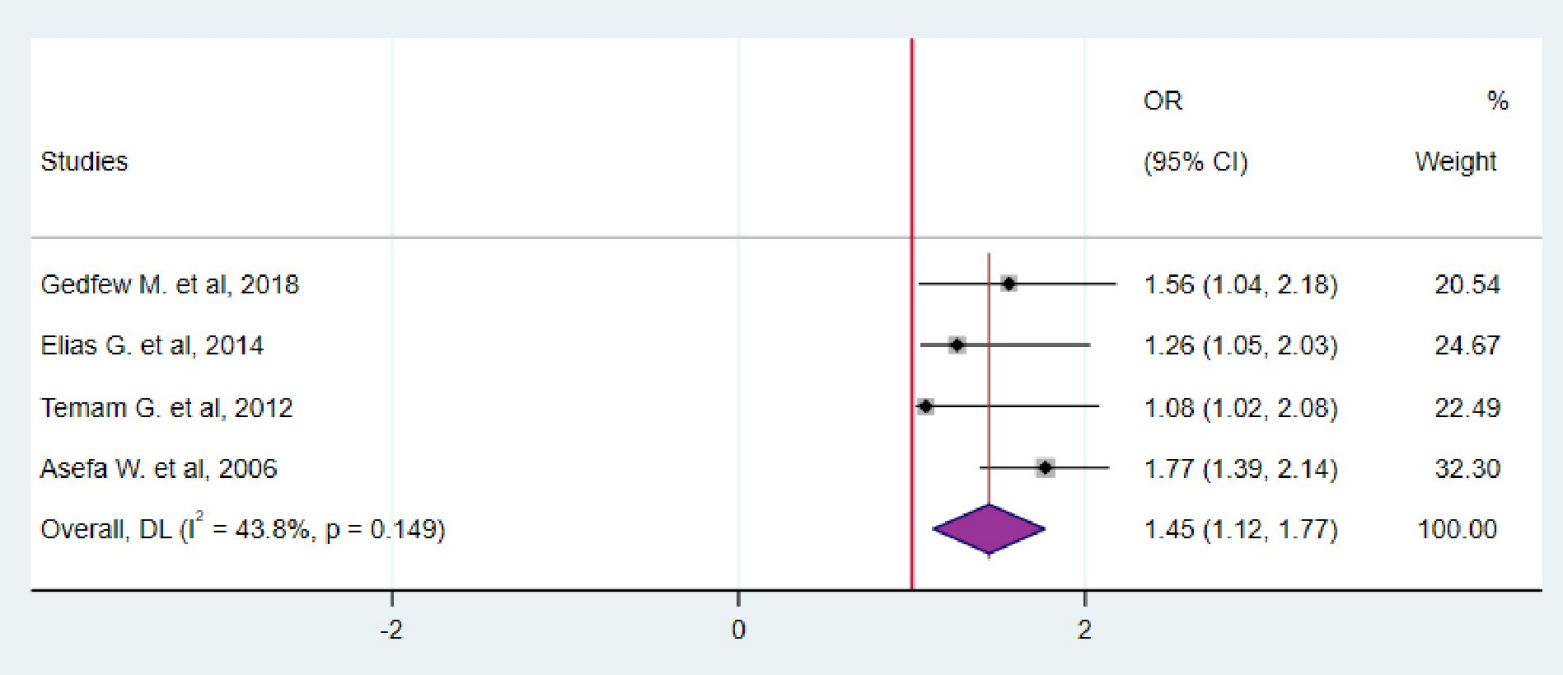
Forest plot for the association between CD4 count and HIV serodiscordance in Ethiopia, 2023.

In addition, the pooled odd ratio for the relationship between CD4 and HIV serodiscordance was evaluated using data from four studies. Accordingly, the findings of this study also showed that those couples whose partner were with CD4 count greater 200 cells/mm^3^ were more likely to be HIV serodiscordant than couples whose either of partner CD4 count less than 200 cells/mm^3^ (OR = 1.45; 95%CI: 1.12–1.77) (Fig 10).

## Discussion

This systematic review and meta-analysis aimed to assess the level of HIV serodiscordance and the factors contributing to its occurrence in Ethiopia. The review provided an overview of the distribution, design, quality, and characteristics of the included studies. A total of ten studies from various regions were included in the analysis, and all of them utilized primary data. It is worth noting that all the studies included in this review were institution-based. The majority of the studies assessed in this review demonstrated good quality. The pooled magnitude of HIV serodiscordance in Ethiopia was found to be 11.4% (95% CI = 7%–16%). This finding is was in line with study conducted in Tanzania, 16% [7] while, lower than studies conducted in some sub-Saharan countries, which revealed the pooled prevalence of HIV serodiscordance among married or cohabited individuals was 23.8% [6], and northern Vietnam, 58% [31]. However, this finding was higher than studies conducted in southeast Asia, china, 2.4% [32] and systematic review conducted in china, 11% [33]. Differences in lifestyle, access to healthcare facilities, public education levels, and cultural and economic backgrounds could all be contributing factors to these differences. Additionally, variations in the approaches used to measure outcome factors in different nations could account for the differences in results.

In our sub-group analysis, we found that a higher level of HIV serodiscordance was observed among individuals who had been on antiretroviral therapy (ART). This finding was supported by a few primary studies conducted in different settings. The variation in HIV serodiscordance rates may be attributed to the biological effects of antiretroviral therapy. There are three mechanisms through which ART can prevent HIV transmission: reduction of HIV viral load in individuals who are aware of their status, post-exposure prophylaxis after risky exposures, and pre-exposure prophylaxis with oral and/or topical microbicides [34].

Additionally, a subgroup analysis was conducted on the serodiscordance of HIV based on the quality of the included studies. The findings revealed that the overall prevalence of HIV serodiscordance was relatively higher among studies of moderate quality compared to those of good quality. Studies with moderate quality may include a broader range of participants, including those from high-risk populations or settings where serodiscordance is more prevalent. High-quality studies often have stricter inclusion criteria, which may result in a more selective sample [33,35].

Furthermore, year of study also subjected to subgroup analysis. Accordingly, among the included studies, the highest pooled prevalence of serodiscordance was observed in studies conducted from 2015 onwards, 17.7% (95% CI: 7%–15.7%). This may be due to advances in HIV testing and diagnostic methods over time may have led to increased detection and identification of serodiscordant individuals. Furthermore, more sensitive and accurate tests could contribute to a higher reported prevalence. Increased awareness about HIV and efforts to promote testing and diagnosis may have led to more individuals getting tested, including those in serodiscordant relationships. This increased testing could result in higher reported prevalence rates.

Couples (or individuals) who regularly used condoms had an 82% higher chance of being HIV serodiscordance than those who did not (OR = 1.82; 95%CI: 1.08–2.56). This could be due to the scarcity of quantitative data supporting the ability of condoms to prevent HIV infection during intercourse. However, regular condom usage is still recommended as one of the primary methods of preventing HIV infection. It is important to note that the consistent use of condoms can effectively prevent the transmission of HIV, with effectiveness ranging from 60% to 70% [36]. This finding consistent with different studies [6,23,37,38].

In addition, the pooled odd ratio for the relationship between CD4 and HIV serodiscordance was evaluated using data from four studies. Accordingly, the findings of this study also showed that those couples whose partner were with CD4 count greater 200 cells/mm^3^ were more likely to be HIV serodicordant than couples whose either of partner CD4 count less than 200 cells/mm^3^ (OR = 1.45; 95%CI: 1.12–1.77). This indicated that low CD4 count show high viral load in serum which directly facilitate high transmission rate from infected individual to uninfected one. This finding is in line with different studies [11,39–42]. Another study conducted in Brazil supported this finding indicating that being HIV detectable viral load will decrease the chance of being serodiscordant [42].

The concept of having an undetectable viral load or a high CD4 count aligns with WHO guidance. The U = U message is supported by strong scientific evidence, which shows that individuals living with HIV who successfully maintain an undetectable viral load through effective antiretroviral therapy (ART) cannot sexually transmit the virus to their partners [43].

Couples (individuals) who had been practicing sexual relationship before marriage were 93% more likely to be serodicordant than their counterparts (OR = 1.93; 95%CI: 1.28–2.57). Couples who engage in premarital sex may not have undergone HIV testing before entering into a sexual relationship. Without knowing their HIV status, individuals are at a higher risk of serodiscordance, as one partner may be living with HIV without being aware of it. This implies that engaging in sexual activity before marriage can increase the likelihood of acquiring HIV from a sexual partner, thereby raising the chances of serodiscordance in a relationship. Compared to married couples, study participants with a history of premarital sex showed a higher likelihood of being serodiscordant. This is consistent with the results of studies from china [32] and an Ethiopian primary investigations [9,23].

### Limitation of the study

This systematic review and meta-analysis possess multiple strengths. It is the first study to consolidate findings from various studies conducted within the country, thereby providing more robust evidence regarding the status of HIV serodiscordance. Moreover, it successfully incorporated a large number of patients (N = 66,534), surpassing the sample sizes of individual studies. Additionally, it attempted to compare the prevalence of HIV serodiscordance across different covariates. Ecological fallacy: Aggregating data from different studies and populations may not always reflect individual-level associations accurately. The findings at the aggregate level may not be applicable or representative for every individual within a specific population. Variations were observed in the measurement techniques used to assess the outcome parameter among the included studies. Additionally, it was not registered on PROSPERO, a prospective registry for systematic reviews and meta-analyses.

## Conclusion

To protect a seronegative partner in a serodiscordant relationship from acquiring HIV infection, it is crucial to implement preventive measures. These measures include providing comprehensive health education on the correct and consistent use of condoms, ensuring regular monitoring and care at an antiretroviral therapy (ART) clinic, and offering voluntary counseling and testing (VCT) services to both sexual partners.

## Supporting information

S1 Checklist PRISMA checklist. (DOCX)

S1 File The bibliographic details of the ten included articles. (CSV)

## References

[1] Al-Jabri AA. Mechanisms of host resistance against HIV infection and progression to AIDS. Sultan Qaboos University Medical Journal. 2007;7(2):82. 21748089 PMC3074872

[2] Organization WH. Focus on key populations in national HIV strategic plans in the WHO African Region. World Health Organization. Regional Office for Africa, 2018.

[3] World Health Organization:Living with HIV when one partner is positive and the other is negative. 2012. https://www.who.int/news-room/feature-stories/detail/living-with-hiv-when-one-partner-is-positive-and-the-other-is-negative.

[4] Curran K, Baeten JM, Coates TJ, Kurth A, Mugo NR, Celum C. HIV-1 prevention for HIV-1 serodiscordant couples. Current HIV/AIDS Reports. 2012;9:160–70. doi: 10.1007/s11904-012-0114-z 22415473 PMC3570050

[5] Carpenter LM, Kamali A, Ruberantwari A, Malamba SS, Whitworth JA. Rates of HIV-1 transmission within marriage in rural Uganda in relation to the HIV sero-status of the partners. Aids. 1999;13(9):1083–9. doi: 10.1097/00002030-199906180-00012 10397539

[6] Gedfew M, Desta M, Mengist B, Amaha H, Hailie D, Bewket B. Prevalence of HIV Sero-discordance among Couples in Sub Saharan Africa, 2019, Systematic Review and Meta Analysis. International Journal of Women’s Health Care. 2020;5(1):18–24.

[7] Ngilangwa DP, Ochako R, Mboya BA, Noronha RH, Mgomella GS. Prevalence and predictors of HIV sero-discordance among cohabiting couples tested in northern Tanzania. Pan African Medical Journal. 2015;22(1). doi: 10.11604/pamj.2015.22.275.5961 26958138 PMC4765341

[8] Gedfew M, Dilie A, Degenu G, Haile D, Yirga T, Bewket B, et al. Incidence and Predictors of Sero-Conversion among HIV Discordant Couples at Amhara Region Selected Public Hospitals, Northwest Ethiopia, 2018: A Mixed Cohort Study. 2021.

[9] Elias Gullma (MD) DEHM, Internist), Dr. Daniel Yilma (MD, Internist), Mr. Dessalegn Massa (BSc, MPH). Sero-conversion rate and its predictors among HIV discordant couples at Jimma University Specialized Hospital. Gray 2014.

[10] Cherinet Y, Berihu A, Bekele A, Biadgilign S, Taye B, Tsegaye A. Trend of HIV prevalence among pregnant women attending Antenatal Care Unit of Bishoftu Hospital, Ethiopia. Ethiop Med J. 2013;51(3):169–76. 24669673

[11] Guthrie BL, Choi RY, Liu AY, Mackelprang RD, Rositch AF, Bosire R, et al. Barriers to Antiretroviral Initiation in HIV-1–Discordant Couples. JAIDS Journal of Acquired Immune Deficiency Syndromes. 2011;58(3):e87–e93. doi: 10.1097/QAI.0b013e31822f064e 21826010 PMC3202340

[12] Bunnell R, Opio A, Musinguzi J, Kirungi W, Ekwaru P, Mishra V, et al. HIV transmission risk behavior among HIV-infected adults in Uganda: results of a nationally representative survey. Aids. 2008;22(5):617–24. doi: 10.1097/QAD.0b013e3282f56b53 18317003

[13] Feyisa GT, Marami SN, Dinagde DD, Dibaba B. Comparative Study of Neonatal Hypothermia and its Associated Factors Among Neonates in Rural and, Urban of Shebadino Woreda, Sidama Region, South Ethiopia: A Community-Based Comparative Cross-Sectional Study. Journal of Clinical Paediatrics and Child Health Care. 2024;1(1). 10.1186/s12889-024-19504-8 PMC11264896 39033283

[14] Colombe S, Beard J, Mtenga B, Lutonja P, Mngara J, de Dood CJ, et al. HIV-seroconversion among HIV-1 serodiscordant married couples in Tanzania: a cohort study. BMC Infectious Diseases. 2019;19(1):518. doi: 10.1186/s12879-019-4151-8 31195994 PMC6567663

[15] Kalichman SC, Rompa D, Luke W, Austin J. HIV transmission risk behaviours among HIV-positive persons in serodiscordant relationships. International journal of STD & AIDS. 2002;13(10):677–82. doi: 10.1258/095646202760326426 12396537

[16] Berhane Y, Mekonnen Y, Seyoum E, Gelmon L, Wilson D. HIV/AIDS in Ethiopia: an epidemiological synthesis. The World Bank, 2008.

[17] Penner M. U = U: The evidence is in. Spreading the word that undetectable = untransmissable is the next crucial step. IDSA. 2021.

[18] [DHHS 2018; CDC 2019] In 2019, The New York State Department of Health AIDS Institute published guidelines for implementing the U = U message in clinical settings, as did the Australasian Society of HIV, Viral Hepatitis and Sexual Health Medicine. [NYDOHAI 2020; ASHM 2020].

[19] World population prospects: UN; 2022. Department of Economic and Social Affairs Population Division https://population.un.org/wpp/FAQs/.

[20] Moher D, Liberati A, Tetzlaff J, Altman DG, Group* P. Preferred reporting items for systematic reviews and meta-analyses: the PRISMA statement. Annals of internal medicine. 2009;151(4):264–9. 19622511 10.7326/0003-4819-151-4-200908180-00135

[21] Singer M, Deutschman CS, Seymour CW, Shankar-Hari M, Annane D, Bauer M, et al. The third international consensus definitions for sepsis and septic shock (Sepsis-3). Jama. 2016;315(8):801–10. doi: 10.1001/jama.2016.0287 26903338 PMC4968574

[22] Desta M, Amha H, Anteneh Bishaw K, Adane F, Assemie MA, Kibret GD, et al. Prevalence and predictors of uterine rupture among Ethiopian women: a systematic review and meta-analysis. PLoS One. 2020;15(11):e0240675. doi: 10.1371/journal.pone.0240675 33137135 PMC7605683

[23] Tadesse M. Assessment of HIV discordance and associated risk factors among couples receiving HIV test in Dilla, Ethiopia. BMC Research Notes. 2014;7(1):893. doi: 10.1186/1756-0500-7-893 25491642 PMC4295257

[24] Habte E, Yami A, Alemseged F, Abdissa Y, Deribe K, Memiah P, et al. Predictors of HIV serodiscordance among couples in southwestern Ethiopia. Journal of the International Association of Providers of AIDS Care (JIAPAC). 2015;14(3):234–40. doi: 10.1177/2325957413488177 23697776

[25] Asefa W. Socio-Demographic and Behavioral Determinants of Sero-Discordance among Couples Taking HIV Test Dessie (Ethiopia): Addis Ababa University; 2006.

[26] Bantigen K, Kitaw L, Negeri H, Kebede M, Wassie A, Bishaw K, et al. Rate of HIV seroconversion among seronegative male partners living with HIV positive women in Addis Ababa, Ethiopia, 2019: A retrospective cohort study. HIV/AIDS-Research and Palliative Care. 2021:125–34. doi: 10.2147/HIV.S281281 33568949 PMC7868707

[27] Temam G, Ali A. Prevalence of HIV and discordant rate and their associated factors among premarital Voluntary Counseling and Testing (VCT) clients in Addis Ababa public VCT centers, Addis Ababa, Ethiopia. Ethiopian Journal of Health Development. 2012;26(3):160–8.

[28] Barnabas G, Pegurri E, Selassie HH, Naamara W, Zemariam S. The HIV epidemic and prevention response in Tigrai, Ethiopia: a synthesis at sub-national level. BMC public health. 2014;14(1):1–11. 24951053 10.1186/1471-2458-14-628 PMC4082278

[29] Teshome GS, Modiba LM. Determinants of mother to child transmission of HIV in Addis Ababa, Ethiopia. International Journal of Africa Nursing Sciences. 2021;15:100348.

[30] Borenstein M, Higgins JP, Hedges LV, Rothstein HR. Basics of meta‐analysis: I2 is not an absolute measure of heterogeneity. Research synthesis methods. 2017;8(1):5–18. doi: 10.1002/jrsm.1230 28058794

[31] Van Tam V, Cuong DD, Alfven T, Phuc HD, Chuc NTK, Hoa NP, et al. HIV sero-discordance among married HIV patients initiating anti-retroviral therapy in northern Vietnam. AIDS Research and Therapy. 2016;13(1):39. doi: 10.1186/s12981-016-0124-9 27891160 PMC5109648

[32] Duan S, Ding Y, Yang Y, Lu L, Sun J, Wang N, et al. Prevalence and correlates of HIV discordance and concordance among Chinese–Burmese mixed couples in the Dehong prefecture of Yunnan province, China. Sexual health. 2012;9(5):481–7. doi: 10.1071/SH12065 23380199

[33] Wang L, Peng Z, Li L, Norris JL, Wang L, Cao W, et al. HIV seroconversion and prevalence rates in heterosexual discordant couples in China: a systematic review and meta-analysis. AIDS care. 2012;24(9):1059–70. doi: 10.1080/09540121.2012.661837 22452488

[34] Gay CL, Cohen MS. Antiretrovirals to prevent HIV infection: pre- and postexposure prophylaxis. Curr Infect Dis Rep. 2008;10(4):323–31. doi: 10.1007/s11908-008-0052-5 . 18765106 PMC3567919

[35] Mashaphu S, Burns JK, Wyatt GE, Vawda NB. Psychosocial and behavioural interventions towards HIV risk reduction for serodiscordant couples in Africa: A systematic review. South African Journal of Psychiatry. 2018;24. doi: 10.4102/sajpsychiatry.v24i0.1136 30263215 PMC6138108

[36] Pinkerton SD, Abramson PR. Effectiveness of condoms in preventing HIV transmission. Soc Sci Med. 1997;44(9):1303–12. doi: 10.1016/s0277-9536(96)00258-4 . 9141163

[37] Were WA, Mermin JH, Wamai N, Awor AC, Bechange S, Moss S, et al. Undiagnosed HIV infection and couple HIV discordance among household members of HIV-infected people receiving antiretroviral therapy in Uganda. JAIDS Journal of Acquired Immune Deficiency Syndromes. 2006;43(1):91–5. doi: 10.1097/01.qai.0000225021.81384.28 16885775

[38] Degefa BD, Feyisa GT, Dinagde DD, Kitil GW, Walle AD. Post-natal care: a vital chance to save mothers and infants! Exploring barriers and factors associated with it: a mixed study. Frontiers in Global Women’s Health. 2023;4. 10.3389/fgwh.2023.1272943 PMC10634507 37954407

[39] Kranzer K, Lawn SD, Johnson LF, Bekker LG, Wood R. Community viral load and CD4 count distribution among people living with HIV in a South African Township: implications for treatment as prevention. J Acquir Immune Defic Syndr. 2013;63(4):498–505. doi: 10.1097/QAI.0b013e318293ae48 . 23572010 PMC4233323

[40] Mbulawa ZZ, Marais DJ, Johnson LF, Boulle A, Coetzee D, Williamson A-L. Influence of human immunodeficiency virus and CD4 count on the prevalence of human papillomavirus in heterosexual couples. Journal of general virology. 2010;91(12):3023–31. doi: 10.1099/vir.0.020669-0 20719990

[41] Djossou SEE, Sossa C, Damien GB, Ahanhanzo-Glèlè R, Tokpanoudé I, Agballa G, et al. Prevalence and Factors Associated with HIV Serodiscordance among Infected Couples in the City of Parakou (Benin). Open Journal of Internal Medicine. 2023;13(4):351–63.

[42] Antonini M, Pontes PS, Melo ES, de Souza Alves R, Gir E, Sorensen W, et al. Serodiscordance predictors among couples in the HIV context: implications for health care. BMC Public Health. 2021;21(1):1849. doi: 10.1186/s12889-021-11835-0 34645401 PMC8513240

[43] World Health Organization: People living with HIV with an undetectable viral load cannot transmit HIV sexually. https://www.who.int/docs/default-source/searo/hiv-hepatitis/joint-moph-unaids-who-uu.pdf?sfvrsn=8378cd0_2.

